# Effects of Fog in Institutions

**Published:** 1914-01-10

**Authors:** 


The Hospital

The Workers' Newspaper of Administrative Medicine and Institutional Life, Administration, National Insurance and Health.

No. 1437, Vol. LV. SATURDAY, JANUARY 10, 1914. PRICE ONE PENNY

### EFFECTS OF FOG IN INSTITUTIONS.

The past year saw an early occurrence of foggy weather. A beautiful autumn, in which early morning sea-bathing was enjoyable even during the first days of October, was characterised by a singular absence of strong winds, and this atmospheric stillness continued in spite of heavy rains. Upon such a meteorological condition fog naturally followed. Notwithstanding the statement of statisticians that the suicide rate for July is the higher, the month of November, with its long nights, its shrouded skies, and murky air, is one of depression to town and country dweller alike; and especially, to those who live and work in institutions, where at all hours of the day and night, be the weather what it may, the busy round goes on. In hospital work, and, indeed, in all medical work, the influence of November weather is speedily felt. Apart from the somewhat mythical cases of such asthmatic individuals as are reported to have found health and comfort in the sulphurous fumes of the old District Railway, the ill-effects of fog are felt alike, though in varying degree, by the infant, the healthy adult, and the emphysematous and bronchitic; amongst the last-mentioned class an actual effect upon the death-rate is observed. Upon the ordinary individual, busy about his affairs, such weather is singularly depressing and irritating; dependent upon artificial light, hampered and delayed, with conjunctivæ and nasal passages smarting and face and clothing besmeared, he acquires a strained condition of temper and a surliness towards his fellows which are assuaged only by the comfort of his fireside.

To the dread of wintry weather is largely due the use of the more complex and artificial methods of ventilation. The processes in use in the House of Commons and in many urban surgical operating theatres and elsewhere, by which air is filtered and warmed and its humidity regulated, are the answer of science to the demand for a cleaner and safer air supply. They are in but a limited degree satisfactory. Air so dealt with is, in a manner curious and as yet imperfectly explained, devitalised; it has very definite disadvantages in sick wards, and produces a train of unpleasant and undesirable symptoms in those exposed continuously to it for many hours.

The climatic conditions of our islands are favourable to the production of mist and fog, often local, very mobile under the influence of wind, and especially prevalent in certain localities over both land and sea. London has gained a notoriety in respect of fogs. Lying on the banks of the Thames, in a basin once marshy and well wooded, surrounded and shut in by hills, river mists have always been frequent. Such mists are common round our coasts, in such places as the Broads of Norfolk, and (even on summer mornings) upon the Upper Thames. In London, as the crowded city grew, the mists acquired a peculiar quality. The smoke resulting from incomplete combustion in domestic and factory fires supplied sooty matter which adhered to the particulate moisture and produced the characteristic black fog, invading the streets or overlying the London basin for hours or days, and producing a blackness dispelled only by rain or wind.

There is a widespread opinion that in London the fogs have of recent years been neither so frequent nor so black. Changing conditions may have tended to limit their visitations. Isolated hamlets, depending for water upon springs or shallow wells, have become connected (with the advent of a cheap and convenient public water supply) by the occupation and drainage of extensive marshy tracts previously waste. As this marvellous growth of Greater London proceeded further large areas underlying the surrounding hills, where the mists were wont to lie, have become covered by buildings; deforestation has gone on; and, with a widespread rain-water catchment, there has been a permanent lowering of the ground-water level throughout the whole London basin. How readily, and even suddenly, ground-water levels, and even local air currents, may be altered is shown by the changes which have resulted in their neighbourhood from the construction of some of the new great water reservoirs to the west of London. Our immense modern London is a warmer, drier city, producing, by heat alone and apart from wind, increased air currents.

Concurrently with some of these changes the present generation has seen the adoption of electric light, the more scientific and economical use of gas, and a marked improvement in the methods of burning coal. With the control of industrial smoke production, the use of stoves allowing of more complete combustion, and possibly the general consumption of a harder quality of coal, the relative smoke output has been in considerable measure reduced.

Are our town fogs, from the bacteriological—the operating-theatre—standpoint, deleterious? Essentially they are not. The water particles, deposited in moisture-saturated air, are in themselves sterile; the sooty matter, also sterile at its birth, contains only some unconsumed hydrocarbons which possess a feeble antiseptic power. The foggy air is, however, a still air, and in that stillness danger lies; organisms of all sorts, with every facility for suspension in the stagnant atmosphere, await the beneficial effects of air-washing rain, of sunlight, and of the oxygenation of movement. If the meteorological conditions primarily responsible for the fogs of our British Islands are beyond our control, we are at least making progress in the art of reducing the more unpleasant of their acquired urban characteristics.

